# Supplementary material for: Quantitative Coronary CT Angiography and Pericoronary Adipose Tissue in Acute Myocardial Infarction: Relationship with Dynamic Myocardial Perfusion SPECT
Source: Diagnostics (Basel). 2025 Nov 9;15(22):2840. doi: 10.3390/diagnostics15222840 (PMC12651576; doi:10.3390/diagnostics15222840)
Supplement: Supplementary file 1 [file diagnostics-15-02840-s001.zip › diagnostics-3955866-supplementary.pdf]

Table S1. Days after AMI at which CCTA and SPECT were Performed

Patient number	Group	CCTA	dynamic SPECT
Patient 1	MINOCA	4	5
Patient 2	MINOCA	8	8
Patient 3	MINOCA	9	5
Patient 4	MINOCA	6	7
Patient 5	MINOCA	5	7
Patient 6	MINOCA	8	8
Patient 7	MINOCA	8	10
Patient 8	MINOCA	6	9
Patient 9	MINOCA	6	6
Patient 10	MINOCA	6	6
Patient 1	MICAD	9	9
Patient 2	MICAD	7	7
Patient 3	MICAD	8	7
Patient 4	MICAD	10	10
Patient 5	MICAD	7	7
Patient 6	MICAD	6	6
Patient 7	MICAD	9	9
Patient 8	MICAD	9	9
Patient 9	MICAD	9	8
Patient 10	MICAD	6	6
Patient 11	MICAD	8	8
Patient 12	MICAD	5	6
Patient 13	MICAD	7	8
Patient 14	MICAD	10	10
Patient 15	MICAD	6	6
Patient 16	MICAD	4	8
Patient 17	MICAD	6	7
Patient 18	MICAD	6	7
Patient 19	MICAD	8	8
Patient 20	MICAD	11	11
Patient 21	MICAD	10	9

CCTA – Coronary computed tomography angiography, MICAD – Myocardial infarction with obstructive coronary artery disease, MINOCA – Myocardial infarction with non-obstructive coronary artery disease, SPECT – Single-photon emission computed tomography
